# Low Vitamin C Concentrations in Patients with Community-Acquired Pneumonia Resolved with Pragmatic Administration of Intravenous and Oral Vitamin C

**DOI:** 10.3390/antiox12081610

**Published:** 2023-08-14

**Authors:** Anitra C. Carr, Emma Vlasiuk, Masuma Zawari, Amy Scott-Thomas, Malina Storer, Michael Maze, Stephen T. Chambers

**Affiliations:** 1 Department of Pathology and Biomedical Science, University of Otago, Christchurch 8011, New Zealand; anitra.carr@otago.ac.nz (A.C.C.); emma.vlasiuk@otago.ac.nz (E.V.); masuma.zawari@otago.ac.nz (M.Z.); amy.scott-thomas@otago.ac.nz (A.S.-T.); 2 Respiratory Services, Christchurch Hospital, Christchurch 4710, New Zealand; malina.storer@cdhb.health.nz (M.S.); michael.maze@cdhb.health.nz (M.M.)

**Keywords:** vitamin C, ascorbic acid, antioxidant, intravenous vitamin C, pneumonia, community-acquired pneumonia, C-reactive protein, procalcitonin, inflammation

## Abstract

Community-acquired pneumonia (CAP) is characterized by elevated markers of inflammation and oxidative stress and depleted circulating concentrations of the antioxidant nutrient vitamin C. A feasibility trial of intravenous and oral vitamin C supplementation, matched to the timing of intravenous and oral antibiotic formulations, was carried out and changes in vitamin C status were monitored to determine whether saturating status could be achieved throughout the administration period. Patients with moderate and severe CAP (CURB-65 ≥ 2; *n* = 75) who were receiving intravenous antimicrobial therapy were randomized to placebo (*n* = 39) or intravenous vitamin C (2.5 g per 8 h; *n* = 36) before moving to oral vitamin C (1 g three times daily) when prescribed oral antimicrobials. Blood samples were collected at baseline and then daily whilst in the hospital. Vitamin C concentrations were determined by high-performance liquid chromatography. The inflammatory and infection biomarkers C-reactive protein and procalcitonin were elevated at baseline (158 (61, 277) mg/L and 414 (155, 1708) ng/L, respectively), and vitamin C concentrations were depleted (15 (7, 25) µmol/L). There was an inverse association between vitamin C and C-reactive protein concentrations (*r* = −0.312, *p* = 0.01). Within one day of intervention initiation, plasma vitamin C concentrations in the vitamin C group reached median concentrations of 227 (109, 422) µmol/L, and circulating concentrations remained at ≥150 µmol/L for the duration of the intervention, whilst median vitamin C concentrations in the placebo group remained low (≤35 µmol/L). There was a trend toward decreased duration of hospital stay (*p* = 0.07) and time to clinical stability (*p* = 0.08) in the vitamin C group. In conclusion, patients with moderate to severe CAP have inadequate plasma vitamin C concentrations for the duration of their hospital stay. The administration of intravenous or oral vitamin C, titrated to match the antimicrobial formulation, provided saturating plasma vitamin C concentrations whilst in the hospital. There were trends toward shorter duration of hospital stay and time to clinical stability. Thus, larger trials assessing the impact of intravenous and oral vitamin C intervention on CAP clinical outcomes are indicated.

## 1. Introduction

Community-acquired pneumonia (CAP) is a severe lower respiratory tract infection characterized by elevated inflammation and oxidative stress [1,2,3]. Vitamin C is an essential dietary nutrient with antioxidant and anti-inflammatory properties and numerous immune-supportive roles [4]. The requirements for vitamin C increase with the severity of infection, whereby critically ill patients with sepsis have the lowest circulating concentrations and approximately 10-fold higher requirements for the vitamin [5,6,7]. Hospitalized patients with CAP typically have depleted vitamin C status (reviewed in [8]), which may be both a consequence and a contributor to the elevated inflammation and oxidative stress observed in this condition [3,9]. There have, however, been relatively few vitamin C intervention studies carried out in adults with pneumonia [10]. Two early oral intervention studies showed decreases in the severity and duration of hospital stays [11,12].

Due to the regulated uptake of oral vitamin C by the intestinal vitamin C transporters, significantly higher circulating concentrations can be achieved via intravenous relative to oral administration of the vitamin [13]. Mahmoodpoor et al. [14] administered placebo or vitamin C as a continuous infusion for 4 days to 80 patients with severe pneumonia and reported improved intensive care parameters in the vitamin C group. Pneumonia is one of the major complications of severe coronavirus disease-2019 (COVID-19), and following the announcement of the SARS-CoV-2 pandemic, there was an upsurge in interest in the efficacy of both intravenous and oral vitamin C administration [15,16]. One of the first trials, from Wuhan, China, in which intravenous placebo or vitamin C was administered every 12 h for 7 days to 54 patients with critical COVID-19, showed decreased markers of inflammation and a trend towards decreased mortality [17].

The current research comprised a substudy nested within a randomised placebo-controlled feasibility trial that used a pragmatic design embedded in clinical practice [18]. The feasibility trial comprised hospitalized patients with moderate and severe CAP (CURB-65 ≥ 2), and intravenous and oral vitamin C supplementation was matched to the severity of the illness as determined by the antimicrobial formulation. Patients who were receiving intravenous antimicrobial therapy were randomized to placebo or intravenous vitamin C (2.5 g per 8 h) before moving to oral vitamin C (1 g three times daily) when they were changed to oral antimicrobial therapy. Due to the limited amount of information available on the efficacy of administering intravenous and oral vitamin C to hospitalised patients with CAP [8], the aim of the substudy was to monitor changes in vitamin C concentrations to determine whether saturating status (plasma concentrations ≥ 70 µmol/L) could be achieved throughout the administration period. Length of hospital stay and time to clinical stability were also monitored.

## 2. Materials and Methods

### 2.1. Participants

The current substudy comprised a prospective analysis of changes in the vitamin C status of the patients following intervention, i.e. a secondary outcome of the main feasibility trial. The feasibility trial was approved by the New Zealand Northern B Health and Disability Ethics Committee (18/NTB/218), and all participants gave written informed consent. The trial was conducted according to the principles of the Declaration of Helsinki and was registered with the Australia New Zealand Clinical Trials Registry (ACTRN12619000256178). Patients with CAP were recruited through Christchurch Hospital, New Zealand, between November 2019 and April 2021. Participants were adults (aged ≥ 18 years) admitted to general medical or respiratory services with CAP, which was defined as an acute illness acquired outside a healthcare setting with clinical features that included increased cough, sputum production, shortness of breath, feverishness, and a new inflammatory infiltrate on chest radiograph. The exclusion criteria were: admission to hospital >48 h prior to screening, unable to give informed consent, CURB-65 pneumonia severity score of <2 [19], pneumonia was not the principal reason for admission, pneumonia associated with bronchial obstruction, bronchiectasis or known tuberculosis, hospital admission in the previous two weeks, severe immunosuppression, history of nephrolithiasis, severe renal impairment, glucose-6-phosphate dehydrogenase deficiency, haemochromatosis, pregnancy, or breastfeeding. All participants tested negative for SARS-CoV-2 prior to being approached.

### 2.2. Sample Size

**Figure 1:**
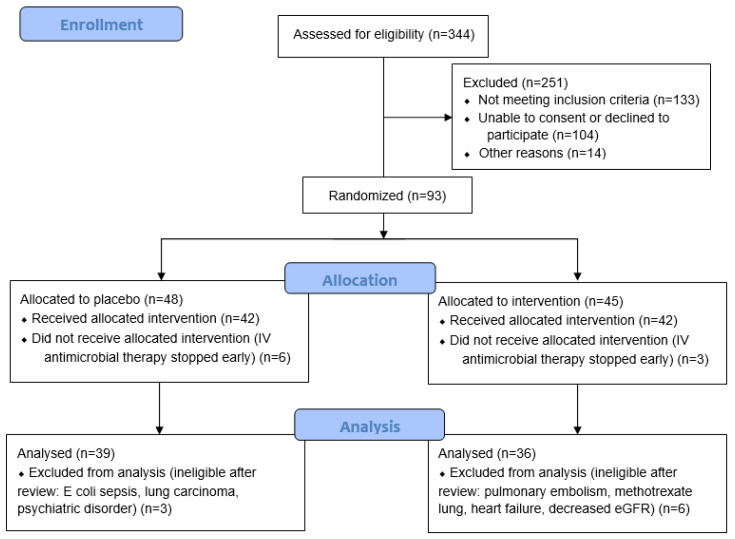
CONSORT study recruitment flow diagram.

Large prospective cohorts (*n* = 718 and 3233) have reported that 50–55% of participants have moderate or severe CAP on hospital admission (CURB-65 of ≥2 or pneumonia severity index (PSI) > 3) [19,20]. There are an estimated 800 CAP admissions to Christchurch Hospital each year. For the primary feasibility outcomes, it was assumed that if at least 25% of the eligible cases were recruited, enrolment rates could exceed 100 participants in a year. This would provide sufficient information to estimate parameter means, variance, and proportions with acceptable precision. Of 344 patients screened for eligibility, 93 were randomised. Patients who were subsequently found to have an alternative diagnosis or were switched to oral antimicrobial therapy before receiving an intravenous dose of study intervention were excluded from the study, resulting in a final cohort of 75 participants (Figure 1).

### 2.3. Randomisation and Masking

Randomisation was carried out by the study biostatistician using a computer-generated list (1:1 allocation). Dispensing of the intravenous and oral preparations was performed by the Christchurch Hospital Pharmacy to ensure those recruiting and enrolling participants and assessing outcomes were blinded to treatment allocation. The interventions were transported and stored in identical opaque packaging and accessed and administered by the nursing staff assigned to care for the patient in the ward. None of the research staff were responsible for administering the interventions.

### 2.4. Intervention

The intravenous interventions (2.5 g vitamin C or normal saline placebo) were added to 100 mL of normal saline, and the infusions were administered over a 20–30 min time period every 8 h until the participants changed from intravenous antimicrobials to oral therapy. The initial intravenous therapy was ASCOR L-500 (McGuff Pharmaceuticals Inc., Santa Ana, CA, USA), which was changed to sodium ascorbate solution (Biological Therapies, Braeside, Australia). The chewable oral tablets (1 g vitamin C or placebo) were administered three times daily. These were sourced from Tishcon Corp, Westbury, NY, USA, and were identical in appearance and flavour.

### 2.5. Study Procedures

The participants’ demographics were obtained at enrolment. The highest CURB-65 score (pneumonia severity) in the 12 h prior to enrolment was recorded [19,20]. Additional clinical data were obtained from electronic hospital records, including time and date of hospital admission and discharge, to determine the length of stay. The time to clinical stability was defined as the time (hours) until stable vital signs for 24 h or longer [21]. Blood samples were collected at hospital admission and daily until discharge.

### 2.6. Biomarker Assessments 

Plasma samples for baseline vitamin C analyses (lithium heparin tubes) were retrieved from the refrigerated (−20 °C) storage facility in the diagnostic laboratory within 6 h [22]. All subsequent samples were collected daily until discharge (excluding weekends). Samples were put immediately on ice, separated within 30 min, and processed for storage at −80 °C. The samples were processed with acid for vitamin C stabilisation and a metal chelator to attenuate oxidation [22]. Analyses were carried out after the completion of the study to maintain blinding. The ascorbic acid concentrations were measured by high-performance liquid chromatography [22]. Saturating vitamin C status was defined as ≥70 µmol/L; adequate, ≥50 µmol/L; inadequate, <50 µmol/L; hypovitaminosis, ≤23 µmol/L; and deficiency, ≤11 µmol/L [23]. The inflammatory and infection biomarkers C-reactive protein and procalcitonin were also assessed; values of >50 mg/L C-reactive protein and ≥250 ng/L procalcitonin indicate likely infection. C-reactive protein was measured by immunoturbidimetry at Canterbury Health Laboratories, an International Accreditation New Zealand (IANZ) laboratory. Procalcitonin was assessed using a commercial ELISA kit (Abcam, Melbourne, Australia). Data were missing for 8 baseline vitamin C samples (3 in the placebo group and 5 in the vitamin C group) and 14 baseline procalcitonin samples (7 in the placebo group and 7 in the vitamin C group).

### 2.7. Statistical Analyses

Median and interquartile range (Q1, Q3) were used for continuous variables, and counts with percentages were used for categorical variables. Group differences were assessed using non-parametric Mann–Whitney U tests, and linear regressions were determined using Pearson’s coefficient. A repeated measures mixed-effects model was used to determine the difference between groups over time. *p* values < 0.05 signified statistical significance. Data analyses and graphical presentations were carried out using GraphPad Prism 9 (GraphPad, San Diego, CA, USA).

## 3. Results

### 3.1. Participant Characteristics

Of the 93 participants recruited for the study, 75 participants were included in the final analysis (Figure 1). Table 1 shows the baseline participant demographics and severity scores, including the data of those randomised to the placebo and vitamin C groups. The median age of the cohort was 76 (70, 83) years, and 42 (56%) were male. The proportion of current smokers was relatively low (<10%). The median CURB-65 score was 3 (2, 3), C-reactive protein was 158 (61, 277) mg/L, and procalcitonin was 414 (155, 1708) ng/L. There were no significant differences between the two randomised groups (*p* > 0.05).

### 3.2. Baseline Plasma Vitamin C Concentrations

**Figure 2:**
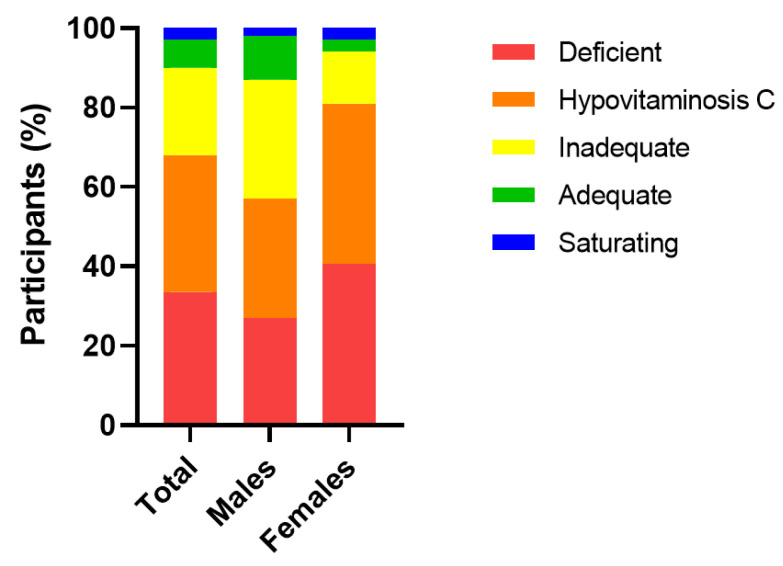
Categories of plasma vitamin C status in the cohort at baseline. Vitamin C categories: deficient, ≤11 µmol/L; hypovitaminosis C, ≤23 µmol/L; inadequate, <50 µmol/L; adequate ≥50 µmol/L; saturating ≥70 µmol/L.

The median (IQR) baseline plasma vitamin C concentration of the cohort was 15 (7, 25) µmol/L (*n* = 67). Of this cohort, 22 (33%) were deficient (i.e., ≤11 µmol/L), 45 (67%) had hypovitaminosis C (i.e., ≤23 µmol/L) and, overall, 60 (90%) had inadequate vitamin C concentrations (i.e., <50 µmol/L). Only 7 (10%) had adequate (≥50 µmol/L) and saturating vitamin C concentrations (≥70 µmol/L). Females had significantly lower median baseline vitamin C status than males (13 (4, 21) µmol/L vs. 20 (11, 36) µmol/L, respectively; *p* = 0.04), and a higher proportion of hypovitaminosis C and deficiency (*p* = 0.08; Figure 2).

**Figure 3:**
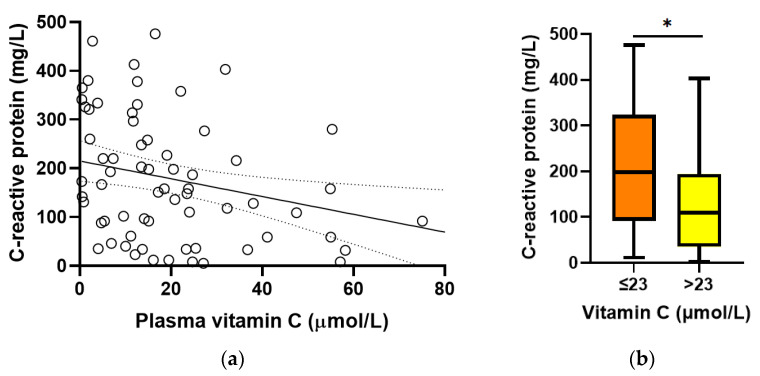
Relationship between baseline vitamin C and C-reactive protein concentrations. (**a**) Linear regression; *r* = −0.312, *p* = 0.01. (**b**) C-reactive protein concentrations relative to hypovitaminosis C cutoff (* *p* = 0.01). Box plots represent the median, with 25th and 75th percentiles as borders, and whiskers represent maximum and minimum values.

Baseline plasma vitamin C concentrations were found to correlate inversely with C-reactive protein concentrations (r = −0.312, *p* = 0.01; Figure 3a). C-reactive protein concentrations were higher in participants with hypovitaminosis C (198 (92, 324) mg/L vs. 110 (35, 194) mg/L, *p* = 0.01; Figure 3b). There was no association between baseline vitamin C and procalcitonin concentrations (r = −0.131, *p* = 0.3).

### 3.3. Plasma Concentrations Achieved with Intravenous and Oral Vitamin C

**Figure 4:**
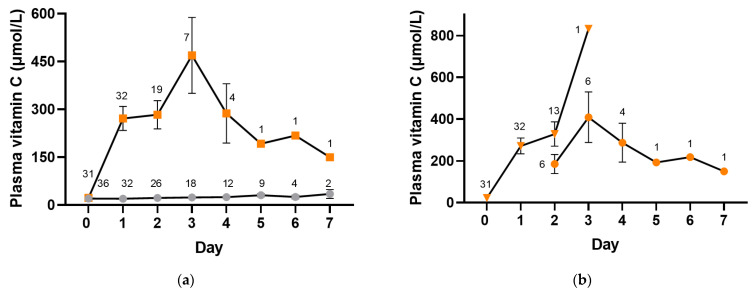
Time course of plasma vitamin C concentrations. (**a**) Placebo group (grey circles) and vitamin C intervention group (orange squares). (**b**) Intravenous vitamin C (orange triangles) and oral vitamin C (orange circles). Intravenous vitamin C (2.5 g per 8 h) was administered for a median of 1 (1, 2) days, and oral vitamin C (1 g three times daily) was administered for a median of 2 (1, 2) days. Data represent mean and SEM, and numbers represent counts.

Participants were administered intravenous placebo or vitamin C (2.5 g per 8 h) whilst receiving intravenous antimicrobial therapy and were changed to oral placebo or vitamin C (1 g three times daily) when prescribed oral antimicrobials. The median (IQR) number of intravenous doses of vitamin C administered was 3 (3, 6) and of placebo was 6 (3, 7). Figure 4 shows plasma vitamin C concentrations in the placebo and intervention groups whilst in hospital (*p* < 0.0001). The median vitamin C concentrations in the placebo group remained low, within the hypovitaminosis C range for the first three days (≤23 µmol/L), and reached a maximum of only 35 (21, 48) µmol/L by day 7. In contrast, the vitamin C group reached concentrations of 227 (109, 422) µmol/L within the first day, with a maximum of 480 (87, 832) µmol/L by day 3. By day 2, approximately one-third of the plasma samples were from participants receiving oral vitamin C, and by day 3, the majority (86%) were from participants receiving oral vitamin C (Figure 4b). Plasma concentrations in the vitamin C intervention group remained at ≥150 µmol/L for the duration of the 7-day intervention.

As can be observed in Figure 4a, fewer participant samples were collected in the vitamin C group from day 2 onwards relative to the placebo group, suggesting the earlier discharge of patients in the vitamin C group. In support of this, time from admission to discharge in the vitamin C group was 69 (48, 116) h vs. 121 (69, 179) h in the placebo group (*p* = 0.07), time from treatment to discharge was 48 (24, 98) hours vs. 98 (46, 165) h in the placebo group (*p* = 0.1), and time from treatment to clinical stability was 22 (−4, 90) h vs. 49 (18, 137) in the placebo group (*p* = 0.08).

## 4. Discussion

Hospitalized patients with CAP generally have low vitamin C status [8], which has been confirmed with the current cohort of moderate to severe patients showing median baseline concentrations in the hypovitaminosis C range (15 (7, 25) µmol/L) as well as a high proportion with deficiency (33%). Although males in the general population tend to have lower vitamin C status than females [24], in the hospitalized patients with CAP, females had lower vitamin C status and a higher proportion of hypovitaminosis C and deficiency than males. The low baseline vitamin C concentrations were associated with elevated inflammation as indicated by an inverse correlation between plasma vitamin C and C-reactive protein; this was not observed in an earlier, less severe cohort [3]. Elevated inflammation is often associated with increased oxidative stress, which is very apparent in hospitalized patients with CAP [3,9]. Oxidative stress can be both a contributor to and a consequence of depleted vitamin C concentrations [8].

It is known that as the severity of an infection increases, so does the demand for vitamin C, with intravenous administration of gram amounts being required in the most severe cases to restore saturating status [5,6,7]. As such, we adopted a pragmatic study design for the feasibility study [18], with intravenous vitamin C being administered (for a median of 1 (1, 2) days) whilst the patients were receiving intravenous antimicrobial therapy during the acute phase of the illness, and oral vitamin C being administered (for a median of 2 (1, 2) days) when the patients had improved and were prescribed oral antimicrobials. Due to the rapid clearance of water-soluble vitamin C by the kidneys, multiple daily doses are recommended to maintain high plasma concentrations [25]. We, therefore, administered both the intravenous and oral vitamin C doses three times daily.

The assessment of plasma concentrations of the vitamin showed saturating concentrations within one day of intravenous vitamin C administration (2.5 g per 8 h). Oral vitamin C administration (1 g three times daily) was able to maintain elevated plasma concentrations (≥150 µmol/L) for the duration of sample collection (7 days) as the illness began to resolve. The resolution was marked by the trend toward clinical stability, which reflects the physiological response to acute infections (body temperature, respiratory rate, blood pressure and confusion). Although peak vitamin C concentrations are relatively transient [25], it is hypothesized that these peak concentrations may still be of benefit through overcoming the downregulation of cellular vitamin C transporters by inflammatory cytokines [26,27].

In contrast to the intervention group, the plasma vitamin C concentrations in the placebo group remained low for the duration of the 7-day study period. This is likely due to a combination of the hospital diet lacking sufficient vitamin C [28] and the greatly enhanced vitamin C requirements of the patients due to their illness. When comparing the outcomes of the two groups, there was a trend towards a decreased length of hospital stay and shorter time to clinical stability in the participants who received vitamin C. Thus, administering sufficient supplemental vitamin C to compensate for the enhanced infection-related demand for the vitamin may help improve the outcomes of CAP patients with depleted baseline vitamin C concentrations. Further dose-finding studies are recommended to elucidate the optimal dosing regimen for these patients.

A limitation of the study included a lower-than-anticipated sample size, primarily due to COVID-related lockdowns and enrolment restrictions, and the exclusion of enrolled participants due to subsequently identified comorbidities, resulting in limited numbers of patients at the longer time points. Plasma samples for biomarker analyses were also not available for all participants at all time points due to a lack of sufficient sample or discharge of the patients to complete their oral intervention at home.

## 5. Conclusions

Overall, low baseline vitamin C concentrations in patients with CAP can be overcome by the administration of intravenous and oral vitamin C matched to the antimicrobial formulation. The trend towards shorter duration of hospital stay and time to clinical stability in those who received supplemental vitamin C suggests larger, fully powered studies are warranted.

## Figures and Tables

**Table 1:** Baseline participant demographics and severity scales.

Characteristics	Total Cohort (*n* = 75)	Placebo Group (*n* = 39)	Vitamin C Group (*n* = 36)
Age, years	76 (70, 83)	77 (70, 83)	76 (70, 85)
Gender, male	42 (56)	18 (46)	24 (67)
Current smoker	7 (9)	4 (10)	3 (8)
CURB-65 score	3 (2, 3)	3 (2, 3)	2.5 (2, 3)
CURB-65 category			
2	36 (48)	18 (46)	18 (50)
3	30 (40)	14 (36)	16 (44)
4	9 (12)	7 (18)	2 (6)
C-reactive protein (mg/L)	158 (61, 277)	158 (62, 282)	145 (60, 257)
Procalcitonin (ng/L) *	414 (155, 1708)	416 (154, 2940)	344 (145, 1484)

Data represent median (Q1, Q2) or *n* (%). CURB-65 score: confusion, blood urea nitrogen, respiratory rate, blood pressure, and age ≥ 65 years. C-reactive protein >50 mg/L and procalcitonin ≥250 ng/L indicate likely infection. * Data were missing for 14 baseline procalcitonin samples (7 in the placebo group and 7 in the intervention group).

## Data Availability

The data presented in this study are available upon reasonable request and at the discretion of the corresponding and first author.
